# HLA-A*33:01 as Protective Allele for Severe Dengue in a Population of Filipino Children

**DOI:** 10.1371/journal.pone.0115619

**Published:** 2015-02-06

**Authors:** Edelwisa Segubre Mercado, Fe Esperanza Espino, Ma. Lucila M. Perez, Josie M. Bilar, Jemimah Dawn P. Bajaro, Nguyen Tien Huy, Benilda Q Baello, Mihoko Kikuchi, Kenji Hirayama

**Affiliations:** 1 Molecular Biology Laboratory, Research Institute for Tropical Medicine, Muntinlupa City, Philippines; 2 Medical Department, Research Institute for Tropical Medicine, Muntinlupa City, Philippines; 3 Office of Research Development, Philippine Children’s Medical Center, Quezon City, Philippines; 4 Department of Immunogenetics, Global Center of Excellence (GCOE) program, Institute of Tropical Medicine (NEKKEN), Graduate School of Biomedical Sciences, Nagasaki University, Nagasaki City, Japan; Tokyo Medical and Dental University, JAPAN

## Abstract

Dengue virus infection is a leading cause of morbidity among children in the Philippines in recent years. In order to investigate the association of HLA Class I and II alleles and dengue disease severity in a cohort of Filipino children, we performed a case control study in 2 hospitals in Metro Manila from June 2008 to December 2009. A total of 250 laboratory confirmed dengue patients and 300 healthy individuals aged 5 to 15 years old were typed for HLA-A, B and DRB1 alleles. The frequency of HLA-A*33:01 was significantly decreased in severe dengue (DHF/ DSS; *Pc* = 0.0016)) and DSS (*Pc* = 0.0032) compared to the background population. These findings support a previous study that this allele may confer protection against the severe form of dengue and provide the first evidence of HLA association with dengue in the Philippines. Future studies should be directed in investigating the possible mechanisms of protection.

## Introduction

Dengue has become the most rapidly spreading mosquito-borne viral disease in the world, with a 30-fold increase in incidence in the last 50 years [1]. The disease is caused by four distinct, but closely related viruses (denoted dengue types 1–4) and can manifest in a range of symptoms, from the mild flu-like illness and rash (dengue fever, DF) to a severe, potentially fatal disease characterized by vascular leakage, hemorrhage, and sometimes hypovolemic shock (dengue hemorrhagic fever, DHF or dengue shock syndrome, DSS) [2]. All four serotypes of dengue virus can cause DF and DHF, with the majority of DHF cases occurring in children [3].

Dengue fever is hyper-endemic in the Philippines, with all four serotypes co-circulating. During the period January 1 to September 7, 2013, the National Epidemiology Center of the Department of Health reported 117, 658 dengue cases from sentinel hospitals nationwide, with 40% of these cases belonging to the 1–10 years old age group [4]. Since the report of the first DHF epidemic in Manila in 1954 [5], epidemics have been occurring in different parts of the country, particularly in Metro Manila and other urban areas.

A central part of the pathogenesis of DHF and DSS is the loss of endothelial integrity, believed to be the result of an abnormal immune response and a disturbance in immune regulation [6]. Although the exact mechanisms leading to these observations remain unclear, host and viral factors have been considered to play a role in progression to severe disease. Epidemiologic and laboratory studies have shown that prior infection with a heterologous viral serotype predisposes progression to DHF, a phenomenon known as antibody-dependent enhancement [7–9]. Viral virulence factors might also determine disease severity: Den-2 was reported to cause more severe infections in Thailand and Vietnam, while more than one serotype was implicated in severe dengue cases in Latin America [10]. Studies on human genetic determinants such as single nucleotide polymorphisms (SNPs) and polymorphisms in the human leukocyte antigen (HLA) genes suggest that they play an important role in susceptibility or protection to dengue disease [6, 11–16]. Although a number of HLA Class I and Class II alleles were found to be associated with DHF/DSS, these might vary according to the study population. To date, there has been no report on the HLA polymorphisms in dengue-infected individuals in the Philippines. This study determined the HLA-A, -B and -DRB1 polymorphisms in Filipino children and their possible association with severity of dengue infections in the country.

## Methods

### Study Population

This study was conducted in two hospitals in Metro Manila, the Philippines: the Research Institute for Tropical Medicine (RITM), located in Alabang, Muntinlupa City, and the Philippine Children’s Medical Center (PCMC) in Quezon City. Suspected dengue patients admitted in these hospitals from June 2008 to December 2009 who satisfied the following criteria were enrolled in the study: age 5 to 15 years old, Filipino Malay race, non-relatedness to other study subjects and no blood transfusion in the past 6 months prior to their admission or consultation to the hospital. Written informed consent was obtained from the parents or legal guardians of all study participants. In addition, assent was also obtained from patients who are 7 years old and above. The study was approved by the institutional review boards of RITM and PCMC.

### Patient clinical data

Medical histories, clinical symptoms and laboratory test results were recorded on case report forms (CRFs). Patients were classified according to disease severity, either as dengue fever (DF), dengue hemorrhagic fever (DHF) or dengue shock syndrome (DSS) based on the established WHO criteria [17] after reviewing the CRFs of the patients. Shock was defined as having pulse pressure of less than or equal to 20mmHg, according to the 2009 WHO guidelines [1]. In addition to the patients’ classification according to established WHO criteria, the presence of warning signs as listed in the 2009 revised dengue classification system were also identified from the data on the CRFs [1]. These warning signs include abdominal pain or tenderness, persistent vomiting, clinical fluid accumulation, mucosal bleed, lethargy/restlessness, liver enlargement >2cm (hepatomegaly) and increase in hematocrit (HCT) concurrent with a rapid decrease in platelet count. Warning signs were defined as follows, which were adopted and modified from Narvaez et al (2011) [18]: Abdominal pain or tenderness: abdominal tenderness or continuous pain (not intermittent), on some occasions diffuse. Persistent vomiting: at least 6 episodes of vomiting in 24 hours, or defined as Grade 3 and above in the Common Toxicity Criteria Manual [19]. Clinical fluid accumulation: pleural effusion and ascites diagnosed clinically or with imaging techniques (ultrasound for ascites, gallbladder wall thickening, and pleural effusion, and/or X-rays for pleural effusion). Mucosal bleeding: bleeding gums or conjunctiva, epistaxis, vaginal bleeding, bleeding from digestive, respiratory or urinary system (kidneys); mucosa defined as respiratory, vaginal, digestive, conjunctival and urinary tract mucosa. Lethargy or restlessness: clinical evaluation of patient’s status. Hepatomegaly: the liver edge palpated by the clinician more than 2 cm below the costal margin. Increased hematocrit concurrent with rapid decrease in platelet count: increase of ≥20% in hematocrit compared to normal values for age and sex [20] concurrent with platelet count ≤100,000 cells/mm^3^. The warning signs were noted anytime during the hospital stay of the patients.

### Sample collection and laboratory diagnosis

Blood samples were collected from patients at the time of study enrolment. Diagnostic tests for dengue virus infection included detection of IgM and IgG antibodies by serology or detection of viral RNA by reverse-transcriptase polymerase chain reaction (RT-PCR). IgM and IgG antibodies were detected in patients’ sera using the PanBio Dengue IgM and IgG Capture Antibody test (Inverness Medical Innovations, Queensland, Australia). The cases were determined as primary infection if the IgM/IgG ratio was greater than 1.78 [21], and consequently, as secondary infection if the ratio was less than 1.78 [22].

Viral RNA was extracted from acute serum samples using QIAamp Viral RNA Mini kit (QIAgen, Hilden, Germany). A widely used hemi-nested RT-PCR assay, developed by Lanciotti et al. [23] targeting the C and pre-M regions of the viral genome, was used for dengue virus detection and serotyping. A modification of this protocol was adopted in this study, which used TS1bis primer instead of TS1 to detect Den-1 [24].

### Population background

Blood samples from healthy, unrelated children from a community in Quezon City and a meningococcemia vaccine efficacy study in Muntinlupa City formed the background population group. The samples were collected specifically for this study, the protocol of which was approved by the institutional review boards of RITM, PCMC and NEKKEN, Nagasaki University. As with the dengue patients, written consent was obtained from parents or legal guardians of all healthy subjects, and assent of children 7 years old and above was also obtained in addition to the consent. The samples from the vaccine study were collected before any vaccine was given to the children. The sera of the background population group were tested for Dengue IgG antibodies using the PanBio Dengue IgG Capture Antibody test (Inverness Medical Innovations, Queensland, Australia).

### HLA typing

For both dengue patient and population background samples, HLA-A, -B and -DRB1 were typed with a microbead sequence-specific oligonucleotide assay (SSO) using LAB type SSO kits (One Lambda, CA, USA) and LAB Scan 100 system (One Lambda, CA, USA). The IS 2.3 typing software and HLA Fusion v.1.2.1 software were used for genotype assignment.

### Statistical analysis

Phenotype frequencies of patient groups were compared pairwise with the population control group to determine possible association. Differences in frequencies were analyzed by the chi-square test or Fisher’s exact test (two-sided) using the StatsDirect statistical software (StatsDirect Ltd., UK), version 2.7.8. The odds ratio (OR) with corresponding 95% Confidence Intervals (95% CI) was computed. P values were corrected using the Bonferroni method, by multiplying with the number of major alleles whose frequencies exceed 5% in either the patient or population group, yielding the corrected P value (Pc). The correction factors were 11 for HLA-A, 18 for HLA-B and 14 for HLA-DRB1. Differences yielding P values or Pc values (for allele frequencies) less than 0.05 were considered significant.

## Results

### Clinical characteristics of the study population

A total of 250 laboratory confirmed dengue patients were included in the study analysis: 220 of these were positive by IgM serology, 132 were positive by RT-PCR and 102 were positive for both tests. The mean age was 9.8, and males comprised 54.4% of patients. The proportion of cases determined as DSS, DHF and DF using the WHO (1997) classification is shown in Table 1. Patients who went into shock (DSS cases) accounted for majority of the patients in this study (122 or 48.8%), followed by DF cases (94 or 37.6%), while DHF cases made up only 13.6% of the patients (Table 1). Most of the patients had secondary infection (78.5%), and the percentage of patients with secondary infection was significantly higher in those with DSS compared to the DF group (OR = 2.65, *P* = 0.005), but was not significant between DSS and DHF, or between the DHF and DF cases.

**Table 1:** Demographic, clinical and laboratory features of dengue patients classified using WHO 1997 classification.

	DSS (n = 122)	DHF (n = 34)	DF (n = 94)	Total (n = 250)	Control (n = 300)
Mean Age (years) ± SD	9.5 ± 2.61	10.6 ± 2.57	10.1 ± 3.05	9.8 ± 2.81	10.0 ± 3.04
Male:Female	64:58	22:12	50:44	136:114	158:142
Secondary infection (%)^a^	95/112 (84.8)	29 (85.3)	59/87 (67.8)	183/233 (78.5)	n.d.
Presence of warning signs (%)					n.d.
Abdominal pain/tenderness^b^	106/121 (87.6)	26 (76.5)	59/93 (64.5)	191/248 (77.0)	n.d.
Persistent vomiting	n.d.^c^	n.d.	n.d.	n.d.	n.d.
Clinical fluid accumulation^b^	52 (42.6)	13 (38.2)	2 (2.1)	67 (26.8)	n.d.
Mucosal bleeding^b^	47 (38.5)	11 (32.4)	19 (20.2)	77 (30.8)	n.d.
Lethargy, restlessness	9 (7.4)	1 (2.9)	2 (2.1)	12 (4.8)	n.d.
Liver enlargement > 2 cm	7/118 (5.9)	1/32 (3.1)	3/92 (3.3)	11/242 (4.5)	n.d.
Increase in hematocrit with rapid decrease in platelet count^b^	29 (23.8)	7 (20.6)	6 (6.4)	42 (16.8)	n.d.
No. of patients positive for serotyping	64	20	46	130	n.d.
Den-1 (%)	3 (4.7)	1 (5)	5 (10.9)	9 (6.9)	n.d.
Den-2 (%)	11 (17.2)	3 (15)	11 (23.9)	25 (19.2)	n.d.
Den-3 (%)	50 (78.1)	15 (75)	30 (65.2)	95 (73.1)	n.d.
Den-4 (%)	0	1 (5)	0	1 (0.8)	n.d.

^a^DSS vs DF, OR = 2.65 (1.3–5.3), *P* = 0.005

^b^DSS vs. DF, OR = 3.89 (2.0–7.7), *P*<0.0001 (Abdominal pain/tenderness); DSS vs. DF, OR = 34.17 (8.0 to 145.1), *P*<0.0001, DHF vs. DF, *P*<0.0001 (Clinical fluid accumulation); DSS vs. DF, OR = 2.47 (1.3–4.6), *P* = 0.0037 (Mucosal bleeding); DSS vs. DF, OR = 4.57(1.8–11.5), *P* = 0.0004, DHF vs. DF, *P* = 0.0405 (Increase in hematocrit with decrease in platelet count)

^c^n.d. = not determined

The most common warning sign in this group of patients was abdominal pain or tenderness (detected in 77% of the patients), which was significantly higher in the DSS group compared to the DF group (OR = 3.89, *P*<0.0001). Likewise, clinical fluid accumulation, mucosal bleeding and increase in hematocrit with rapid decrease in platelet count were significantly higher in DSS compared to the DF group (Table 1). On the other hand, only 11 out of 242 patients had liver enlargement > 2cm or hepatomegaly (4.5%, Table 1) in this study population.

Den-3 was the predominant serotype (73.1%), followed by Den-2 (19.2%) and Den-1 (6.9%, Table 1). However, infection with a particular serotype was not associated with disease severity in this study (data not shown).

The mean age of the background population group was 10.0, with males accounting for 53% of the subjects (Table 1). Moreover, eight out of 300 (2.7%) healthy subjects were seropositive for dengue IgG antibodies.

### Phenotype frequencies of HLA-A, HLA-B and HLA-DRB1

The major alleles in this study, defined as alleles with more than 5% phenotype frequencies in either the patient or population background group, are as follows: HLA-A* 02:01, 02:03, 02:06, 02:27, 11:01, 24:02, 24:03, 24:07, 24:10, 33:01, 34:01; HLA-B* 07:05, 15:01, 15:02, 15:07, 15:13, 15:21, 15:35, 18:01, 35:01, 35:05, 38:02, 40:01, 40:02, 46:01, 48:01, 51:01, 58:01; HLA-DRB1* 03:01, 04:03, 04:05, 04:07, 04:10, 07:01, 08:03, 09:01, 11:01, 12:02, 14:01, 14:05, 15:01, 15:02.

### Association of HLA Alleles with Severe Dengue

The phenotype frequencies of HLA-A, HLA-B and HLA–DRB1 in the severe dengue (DSS and DHF), DF and background population groups are listed in Table 2. HLA-A* 33:01 was associated with a reduced risk of developing severe dengue (DSS/DHF) when compared to the background population (OR = 0.2, *Pc* = 0.0022). The HLA-B alleles 07:05, 38:02 and 58:01 and HLA-DRB1* 03:01 were also reduced in the severe dengue group compared to the population background group, but these were not statistically significant after correction. On the other hand, HLA-B alleles 35:01 and 51:01 and HLA-DRB1* 12:02 were associated with a higher risk of developing severe dengue compared to the background population. However, these alleles also did not withstand correction. When comparing DF to the population background group, HLA-A *02:03 was increased in the patient group, while HLA-B *48:01 and HLA-DRB1* 03:01 were reduced in the patient group, but again, these were not statistically significant.

**Table 2:** Phenotype frequencies of HLA-A, HLA-B and DRB1 and their association with dengue disease severity.

				DSS and DHF vs Control			DF vs Control			DSS and DHF vs DF		
Phenotype	DSS and DHF No. (%)	DF No. (%)^a^	Control No. (%)^b^	OR (95% CI)	P value	Pc^b^	OR (95% CI)	P value	Pc	OR (95% CI)	P value	Pc
A locus	n = 153	n = 92	n = 296									
02:03	2 (1.3)	5 (5.4)	3 (1.0)	1.3 (0.2–7.8)	>0.999	-	5.6 (1.3–24)	0.021	0.231	0.2 (0.04–1.2)	0.107	-
33:01	3 (2.0)	5 (5.4)	34 (11.5)	0.2 (0.05–0.5)	0.0002	0.0022	0.4 (0.2–1.2)	0.086	-	0.3 (0.08–1.5)	0.156	-
B locus	n = 155	n = 93	n = 297									
07:05	3 (1.9)	4 (4.3)	18 (6.1)	0.3 (0.09–1.1)	0.0439	0.790	0.7 (0.2–2.1)	0.551	-	0.4 (0.1–2.0)	0.311	-
35:01	23 (14.8)	7 (7.5)	24 (8.1)	2.0 (1.1–3.6)	0.03	0.540	0.9 (0.4–2.2)	0.890	-	2.1 (0.9–5.2)	0.09	-
38:02	34 (21.9)	24 (25.8)	91 (30.6)	0.6 (0.4–1.0)	0.0489	0.880	0.8 (0.5–1.3)	0.378	-	0.8 (0.4–1.5)	0.489	-
48:01	11 (7.1)	2 (2.2)	24 (8.1)	0.9 (0.4–1.8)	0.7257	-	0.3 (0.06–1.1)	0.0365	0.657	3.5 (0.8–16.0)	0.0935	-
51:01	16 (10.3)	6 (6.5)	15 (5.1)	2.2 (1.0–4.5)	0.0425	0.765	1.3 (0.5–3.4)	0.595	-	1.7 (0.6–4.4)	0.312	-
58:01	5 (3.2)	4 (4.3)	28 (9.4)	0.3 (0.1–0.8)	0.0131	0.236	0.4 (0.1–1.3)	0.112	-	0.7 (0.2–2.8)	0.667	-
DRB1 locus	n = 153	n = 90	n = 300									
03:01	4 (2.6)	2 (2.2)	24 (8)	0.3 (0.1–0.9)	0.0204	0.286	0.3 (0.06–1.1)	0.0447	0.626	1.2 (0.2–6.6)	0.886	-
12:02	56 (36.6)	27 (30)	79 (26.3)	1.6 (1.1–2.5)	0.0256	0.358	1.2 (0.7–2.0)	0.493	-	1.3 (0.8–2.4)	0.299	-

^a^The number for each HLA locus shows the number of successfully typed samples

^b^Corrected P-values (Pc) were only calculated for loci with P-values less than 0.05

### Association of HLA Alleles with the development of shock

Table 3 shows the phenotype frequencies of patients which developed shock (DSS), those with severe dengue but without shock (DHF) and the background population group. HLA-A* 33:01 was significantly reduced in dengue patients which developed shock compared to the background population (OR = 0.1, *Pc* = 0.0044). The HLA-B alleles 38:02, 58:01 and DRB1 allele 03:01 were reduced in the DSS group compared to the population background, but this was not statistically significant after correction. HLA-B* 35:01 was associated with a higher risk of developing DSS compared to the background population; however, this was also not statistically significant.

**Table 3:** Phenotype frequencies of HLA-A, HLA-B and DRB1 and their association with presence or absence of shock in severe dengue.

				DSS vs DHF			DSS vs Control			DHF vs Control		
Pheno-type	DSS No.^a^ (%)	DHF No.^a^ (%)	Control No.^a^ (%)	OR (95% CI)	P value	Pc^b^	OR (95% CI)	P value	Pc	OR (95% CI)	P value	Pc
A locus	n = 120	n = 34	n = 296									
33:01	2 (1.7)	1 (2.9)	34 (11.5)	0.6 (0.05–6.4)	0.530	-	0.1 (0.03–0.6)	0.0004	0.0044	0.2 (0.03–1.8)	0.116	-
B locus	n = 121	n = 34	n = 297									
35:01	21 (17.4)	2 (5.9)	24 (8.1)	3.4 (0.7–15.1)	0.093	-	2.4 (1.3–4.5)	0.008	0.144	0.7 (0.2–3.1)	P>0.99	-
38:02	25 (20.7)	9 (26.5)	91 (30.6)	0.7 (0.3–1.7)	0.475	-	0.6 (0.4–1.0)	0.0375	0.675	0.8 (0.4–1.8)	0.634	-
58:01	4 (3.3)	1 (2.9)	28 (9.4)	0.5 (0.1–3.1)	0.613	-	0.3 (0.1–1.0)	0.0273	0.491	0.3 (0.04–2.2)	0.336	-
DRB1 locus	n = 120	n = 33	n = 300									
03:01	3 (2.5)	3 (3.0)	24 (8)	0.8 (0.08–8.2)	>0.99	-	0.3 (0.09–1.0)	0.0315	0.441	0.4 (0.05–2.7)	0.490	-

^a^The number for each locus shows the number of successfully typed samples

^b^Corrected P-values (Pc) were only calculated for loci with P-values less than 0.05

## Discussion

Dengue has become the most important vector-borne disease in the Philippines, but there is little published data on the clinical characteristics of the disease, and none on host factors affecting this disease in the population. This study describes the clinical characteristics of dengue patients aged 5 to 15 years old in 2 hospitals in Metro Manila from June 2008 to December 2009, and presents the first evidence of possible genetic factors influencing disease severity in the Filipino population.

Majority of the patients in the study developed shock (DSS, 48.8%), followed by mild DF (37.6%) and DHF but without shock (13.6%; Table 1). This is in contrast to an earlier study done in 1999–2001 in another hospital in Metro Manila, in which most of the patients had DF (66.6%), and of those that developed DHF, only 3/120 patients were classified as DHF III and IV [25]. The 7–10 year time difference between study periods and the hospital setting may have contributed to the difference in proportions of severe dengue between our studies. The number of dengue cases has risen more than 500% from 1999 to 2009, with a corresponding ~300% increase in deaths during this period [26]. In addition to the rise in the number of cases, the earlier study was also conducted in a private hospital where dengue patients are seen earlier, while our study was conducted in 2 government referral hospitals where patients are usually admitted when they are in DHF stage 1 or 2, and DF cases are mostly seen in the outpatient department. Nevertheless, even with this difference, abdominal pain is an important finding in both of our studies, and it was also increased in patients with the more severe form of dengue. Although the pathogenesis of abdominal pain in dengue is not clearly understood, previous studies have implicated hepatitis, acalculous cholecystitis, acute pancreatitis and hepatomegaly among other causes [27–28]. Hepatomegaly is a significant finding in other Asian patients with DHF or DSS [12, 29–30], but is present in only a small proportion in our study population (4.5%, Table 1). Reports from early dengue outbreaks in the Philippines in 1966 and 1983 also showed a similar low level of hepatomegaly in the patients studied [31–32]. These findings suggest that hepatomegaly is not a major contributor to abdominal pain in our study. As abdominal pain is a consistent finding in dengue patients in the Philippines, future studies should investigate the possible causes of this further.

In this study, under the clinical setting of increasing number of severe cases, we aimed to identify HLA A, B and DRB1 alleles that are associated with development of dengue in a population of Filipino children living in 2 cities in Metro Manila, the urban capital of the Philippines. We compared the allele frequencies of patients with severe dengue (DHF/DSS), dengue patients with shock (DSS) and those with mild dengue (DF) to age-matched background population who never had symptomatic dengue. In this background population, 8/300 (2.7%) were seropositive to dengue IgG antibodies at the time of the study. Our results present the first evidence of host genetic factors which may be associated with protection against dengue in the Filipino population. Various studies have implicated HLA Class I and II alleles which are associated with susceptibility and protection against severe forms of dengue [13–15]. In our study, HLA-A*33:01 conferred protection against severe dengue, as shown by the decreased frequency of this allele in patients which developed severe dengue (DHF/DSS) compared to the background population (OR = 0.2, *Pc* = 0.0022) as well as in patients who developed shock compared to the background population (OR = 0.6, *Pc* = 0.0044). Even the non-severe dengue group (DF) showed a tendency to be decreased in their HLA-A*33:01 frequency compared to the background population. Loke *et al* also reported that Vietnamese children with HLA-A*33 were less likely to develop DHF [16]. Recently, a similar tendency was also observed by Appanna *et al* in the Indian DHF patient group in Malaysia [33], although this was not statistically significant.

When HLA-A*33:01 was analyzed for linkage disequilibrium with alleles in other loci found in this study to have a similar tendency in dengue patients (i.e., protection against severe dengue), we found that HLA-B*58:01 and HLA-DRB1*03:01 are in linkage disequilibrium with A*33:01 (data not shown). The A*33-B*58 and A*33-DRB1*03 haplotypes were previously reported to be common in a minority population in Inner Mongolia [34]. Since the association of dengue with B*58:01 and DRB1*03:01 alleles in this study was lost after Bonferroni correction, the association is probably due to A*33:01, which remained even after correction. A33 belongs to the HLA-A3 supertype family, which includes the allelic products of at least 5 common HLA-A alleles, A3, A11, A31, A33 and A68. These alleles are grouped based on structural similarities and peptide binding motif analysis [35]. However, we did not find an association between the other HLA-A3 supertype alleles and dengue in this study. Dengue-specific peptides that restrict HLA-A33 have been identified; a possible mechanism of protection for CD8 T-cells, which were shown to be restricted by this allele, is to limit viral infection by cytolysis of infected cells and secretion of antiviral cytokines [16].

Although HLA-A* 33:01 was the only allele which reached statistical significance after correction in this study, other alleles were also found to either increase or decrease in the patient groups compared to the background population group, but were not significant after correction. HLA-A*02:03 increased in frequency in DF compared to the background population group (OR = 5.6, *P =* 0.021). This allele was previously reported by Stephens *et al* to be increased in frequency in DF patients with secondary dengue infections when compared to either controls or DHF patients with secondary infections in Thailand [36]. In this study, HLA-B*07:05 was associated with a reduced risk of developing severe dengue (DHF/DSS), HLA-B alleles 38:02 and 58:01 were associated with a reduced risk of developing both severe dengue (DHF/DSS) and DSS, and HLA-B*48:01 was reduced in the DF compared to the background population group. HLA-B*07 was also found at a very low frequency in patients with shock compared to those who did not develop shock and the normal population in Sri Lanka [37] and B*58 was found at lower frequencies in DHF patients of Malay, Chinese and Indian origins in Malaysia [38], although this was not statistically significant in both studies. However, in contrast, HLA-B*48 was detected in higher frequencies in Malay and Chinese DHF patients in the same study in Malaysia, although this was also not significant [38]. Our study also found HLA-B*35:01 to be increased in the severe dengue and DSS groups, and HLA-B*51:01 to be increased in the severe dengue group compared to the background population. The role of HLA-B*35:01 in dengue is uncertain as differing associations were found in previous reports. Brown *et al* (2011) reported a statistically significant increase in B*35 dengue cases compared to controls in a Jamaican population, although this finding did not remain significant after correction [39]. In contrast, a study on Mexican Mestizo patients found that B*35 was negatively associated with symptomatic dengue and DF [40]. HLA-B*51 was also associated with the development of DHF in ethnic Thai patients with secondary dengue infections [36]. Two HLA Class II alleles, HLA-DRB1*03:01 and DRB1*12:02, were also associated with dengue in our study population, although the significance was lost after correction. The frequency of DRB1*03:01 was decreased in all the dengue groups- severe dengue (DHF/DSS), DSS and DF, when compared to the background population, while DRB1*12:02 was associated with a higher risk of developing severe dengue (OR = 1.6, *P =* 0.0256). As mentioned earlier, DRB1*03:01 has significant linkage with A*33:01. Malavige *et al* (2011) reported an association of DRB1*12 allele with the development of DHF during primary dengue infections in Sri Lanka [37]. Although the associations of these HLA Class I and II alleles to dengue were lost after Bonferroni correction, most of these alleles were previously reported to be associated with the development or protection to dengue disease. Since it is possible that the loss of significance may be a consequence of statistical analysis, additional studies with larger sample sizes are necessary in order to clarify their role in susceptibility or protection in dengue disease.

The results of our study showed HLA-A*33:01 as a possible protective allele for severe dengue, which supports previous reports of host genetic factors affecting disease severity. Other factors have also been implicated in disease severity, including secondary dengue virus infection and the infecting viral serotype. In our study, majority of our patients (78.5%) had secondary dengue infection based on IgG/IgM ratio. We found that a significantly higher proportion of patients with DSS had secondary infection compared to DF patients (OR = 2.65, *P =* 0.005). This is consistent with studies that correlate secondary infection and disease severity [8–9, 41]. However, we did not find any difference between DSS and DHF patients, or between DHF and DF. During the study period, 2008 to 2009, we detected all 4 dengue serotypes, with Den-3 as the predominant circulating serotype (73.1%), followed by Den-2 (19.2%). We were able to amplify and subsequently obtain serotype data for 52% of our patients, which is relatively high, considering that most of the blood samples were collected after Day 5 of illness. Although previous reports have correlated disease severity and viral serotype [10, 41], we did not find any association between the infecting serotype and disease severity in our study.

In conclusion, our study supports previous findings implicating HLA-A*33:01 in protection against the severe form of dengue, and presents the first evidence of HLA association with dengue in the Philippines. We recommend future studies to investigate possible mechanisms of protection in order to contribute to a better understanding of dengue immunopathogenesis.

## Supporting Information

S1 Table Phenotype frequencies of major HLA-A, HLA-B and HLA-DRB1 alleles in the study population. (DOCX)
