# Supplementary material for: HLA-A*33:01 as Protective Allele for Severe Dengue in a Population of Filipino Children
Source: PLoS One. 2015 Feb 6;10(2):e0115619. doi: 10.1371/journal.pone.0115619 (PMC4319754; doi:10.1371/journal.pone.0115619)
Supplement: S1 Table — (DOCX) [file pone.0115619.s001.docx]

Table S1. Phenotype frequencies of major HLA-A, HLA-B and HLA-DRB1 alleles in the study population.

| **Phenotype** | **DSS No. (%)^a^** | **DHF No. (%)** | **DF No. (%)** | **Control No. (%)** |
| --- | --- | --- | --- | --- |
| **A locus** | n=120 | n=34 | n=92 | n=296 |
| 02:01 | 22 (18.3) | 4 (11.8) | 10 (10.9) | 38 (12.8) |
| 02:03 | 2 (1.7) | 0 | 5 (5.4) | 3 (1.0) |
| 02:06 | 4 (3.3) | 3 (8.8) | 2 (2.2) | 14 (4.7) |
| 02:27 | 0 | 2 (5.9) | 0 | 1 (0.3) |
| 11:01 | 33 (27.5) | 9 (26.5) | 25 (27.2) | 99 (33.4) |
| 24:02 | 32 (26.7) | 11 (32.4) | 28 (30.4) | 82 (27.7) |
| 24:03 | 21 (17.5) | 7 (20.6) | 17 (18.5) | 50 (16.9) |
| 24:07 | 34 (28.3) | 7 (20.6) | 24 (26.1) | 64 (21.6) |
| 24:10 | 4 (3.3) | 0 | 8 (8.7) | 10 (3.4) |
| 33:01 | 2 (1.7) | 1 (2.9) | 5 (5.4) | 34 (11.5) |
| 34:01 | 38 (31.7) | 10 (29.4) | 31 (33.7) | 91 (30.7) |
| **B locus** | n=121 | n=34 | n=93 | n=297 |
| 07:05 | 2 (1.7) | 1 (2.9) | 4 (4.3) | 18 (6.1) |
| 15:01 | 9 (7.4) | 3 (8.8) | 8 (8.6) | 27 (9.1) |
| 15:02 | 5 (4.1) | 1 (2.9) | 4 (4.3) | 15 (5.1) |
| 15:07 | 5 (4.1) | 2 (5.9) | 4 (4.3) | 10 (3.4) |
| 15:13 | 8 (6.6) | 2 (5.9) | 6 (6.5) | 11 (3.7) |
| 15:21 | 15 (12.4) | 4 (11.8) | 8 (8.6) | 31 (10.4) |
| 15:35 | 8 (6.6) | 3 (8.8) | 4 (4.3) | 18 (6.1) |
| 18:01 | 3 (2.5) | 3 (8.8) | 5 (5.4) | 21 (7.1) |
| 35:01 | 21 (17.4) | 2 (5.9) | 7 (7.5) | 24 (8.1) |
| 35:05 | 19 (15.7) | 6 (17.6) | 13 (14.0) | 38 (12.8) |
| 38:02 | 25 (20.7) | 9 (26.5) | 24 (25.8) | 91 (30.6) |
| 40:01 | 16 (13.2) | 7 (20.6) | 17 (18.3) | 61 (20.5) |
| 40:02 | 16 (13.2) | 3 (8.8) | 13 (14.0) | 26 (8.8) |
| 46:01 | 2 (1.7) | 2 (5.9) | 7 (7.5) | 12 (4.0) |
| 48:01 | 10 (8.3) | 1 (2.9) | 2 (2.2) | 24 (8.1) |
| 51:01 | 10 (8.3) | 6 (17.6) | 6 (6.5) | 15 (5.1) |
| 51:06 | 4 (3.3) | 2 (5.9) | 3 (3.2) | 12 (4.0) |
| 58:01 | 4 (3.3) | 1 (2.9) | 4 (4.3) | 28 (9.4) |
| **DRB1 locus** | n=120 | n=33 | n=90 | n=300 |
| 03:01 | 3 (2.5) | 1 (3.0) | 2 (2.2) | 24 (8) |
| 04:03 | 6 (5) | 4 (12.1) | 7 (7.8) | 23 (7.7) |
| 04:05 | 17 (14.2) | 5 (15.2) | 17 (18.9) | 41 (13.7) |
| 04:07 | 0 | 2 (6.1) | 0 | 1 (0.3) |
| 04:10 | 1 (0.8) | 2 (6.1) | 2 (2.2) | 2 (0.7) |
| 07:01 | 8 (6.7) | 2 (6.1) | 7 (7.8) | 28 (9.3) |
| 08:03 | 5 (4.2) | 1 (3.0) | 5 (5.6) | 13 (4.3) |
| 09:01 | 14 (11.7) | 4 (12.1) | 9 (10) | 48 (16) |
| 11:01 | 19 (15.8) | 3 (9.1) | 10 (11.1) | 28 (9.3) |
| 12:02 | 41 (34.2) | 10 (30.3) | 27 (30) | 79 (26.3) |
| 14:01 | 3 (2.5) | 3 (9.1) | 2 (2.2) | 7 (2.3) |
| 14:05 | 0 | 3 (9.1) | 1 (1.1) | 2 (0.7) |
| 15:01 | 41 (34.2) | 11 (33.3) | 33 (36.7) | 107 (35.7) |
| 15:02 | 53 (44.2) | 12 (36.4) | 44 (48.9) | 142 (47.3) |

^a^The number for each HLA locus shows the number of successfully typed samples
